# Is small size at birth associated with early childhood morbidity in white British and Pakistani origin UK children aged 0–3? Findings from the born in Bradford cohort study

**DOI:** 10.1186/s12887-018-0987-0

**Published:** 2018-02-01

**Authors:** Jane West, Brian Kelly, Paul J. Collings, Gillian Santorelli, Dan Mason, John Wright

**Affiliations:** 1 Bradford Institute for Health Research, Bradford Royal Infirmary, Duckworth Lane, Bradford, BD9 6RJ UK; 2 School of Social & Community Medicine, University of Bristol, Bristol, UK

## Abstract

**Background:**

Low birthweight is associated with increased infant morbidity, poorer developmental outcomes and risk of adult disease and its prevention remains a public health priority. South Asian children are more likely to be born small and there is some debate around whether this is a normal phenomenon within this population or whether they have a greater risk of morbidity. We examined the association between small size at birth and morbidity in White British and Pakistani origin children aged 0–3 participating in the Born in Bradford cohort study.

**Methods:**

We included 4119 White British and 4731 Pakistani origin children and examined health service use (General Practitioner (GP) consultations, the most common prescribing categories (analgesics, antibiotics, bronchodilators), emergency and elective hospital episodes) as markers of morbidity, in children born small defined as: (i) low birthweight (< 2500 g) (ii) small for gestational age (SGA) using customised birth charts. We used negative binomial regression models to predict the adjusted incidence of morbidity markers.

**Results:**

8.7% of Pakistani and 5% of White British children were born with a low birthweight (< 2500 g). Using customised charts, these proportions were 15.3 and 6.2% respectively. Children born small in both groups irrespective of the criteria used, generally had a higher rate of episodes for morbidity markers compared to normal weight children. Incidence of GP consultations (IRR 1.48 (95% CI 1.27, 1.73) to 1.55 (95% CI 1.36, 1.76) depending on birthweight category), analgesic (IRR 1.76 (95% CI 1.37, 2.25) to 2.31 (95% CI 2.19, 2.45) and antibiotic prescriptions (IRR 1.13 (95% CI 0.08, 1.46) to 1.38 (95% CI 1.30, 1.48) and emergency hospital episodes (IRR 1.20 (95% CI 1.06, 1.36) to 1.46 (95% CI 0.92, 2.32), was higher in Pakistani origin children with either a low or normal birthweight.

**Conclusion:**

Being born small is associated with greater morbidity estimated by use of health services, in both White British and Pakistani origin children underlining the importance of public health policy to reduce low birthweight. Pakistani origin children access health services more frequently than White British children irrespective of birthweight and this has implications for health service planning in areas with South Asian populations.

## Background

Birthweight reflects intrauterine growth and wellbeing and is recognised globally as an indicator of infant, child health and increasingly adult health [1]. A low birthweight (< 2500 g) has previously been associated with increased infant mortality and morbidity [2, 3], poorer education outcomes and developmental delay in childhood [4, 5] and an increased risk of adult disease [6] via a range of programming mechanisms. It has been suggested that one such mechanism is the potential for reduced immune function [7] and an inverse association between birthweight and infection related morbidity has been identified in children up to the age of 14 [8], and there is some evidence of longer term effects of birthweight on the immune system where antibody response to vaccination in teenagers and adults is lower in those who were small at birth [9, 10]. Worldwide mean birthweight is lower and the incidence of low birthweight higher among South Asian origin populations when compared to White US/European populations [11, 12]. In South Asia, this is in part thought to reflect environmental exposure to poverty and poor nutrition. However, babies born in high income countries such as the UK, to mothers of South Asian origin are considerably lighter (around 200-300 g) than babies born to White British mothers and this difference does not appear to reduce over subsequent generations of UK South Asians [13, 14]. This has led to the suggestion that differences may not be expressions of growth restriction but rather are genetically or culturally programmed [15–17], and in the absence of environmental risk factors, may not necessarily lead to increased mortality or morbidity i.e. some South Asian infants may be small and healthy rather than small and at risk. Whether it can be proven that smaller size is a normal phenomenon within South Asians or not, the important question is whether the risks of greater mortality or morbidity arising from smaller birth size still apply. Customised growth charts have been developed that take into account ethnicity and other maternal factors [15] but when these have been evaluated in terms of neonatal risk, there is no strong evidence that these better predict the risk of adverse outcomes than population based charts [18]. Whether this is also the case beyond the neonatal period, to our knowledge remains unclear.

Our aim in this paper was to examine the association between being born small and early childhood morbidity estimated using use of health services information for White British and Pakistani origin children aged 0–3 participating in the Born in Bradford (BiB) cohort study. We chose GP prescribing as a marker of morbidity and selected the three most common prescribing categories: analgesics, antibiotics and bronchodilators, where analgesics was the most common category followed by antibiotics and third, bronchodilators. We also considered the number of GP consultations and emergency and elective hospital episodes as further morbidity indicators. We defined being born small in two ways using a cut-off of being born weighing less than 2500 g and also using customised birthweight charts.

## Methods

### Population

**Fig. 1:**
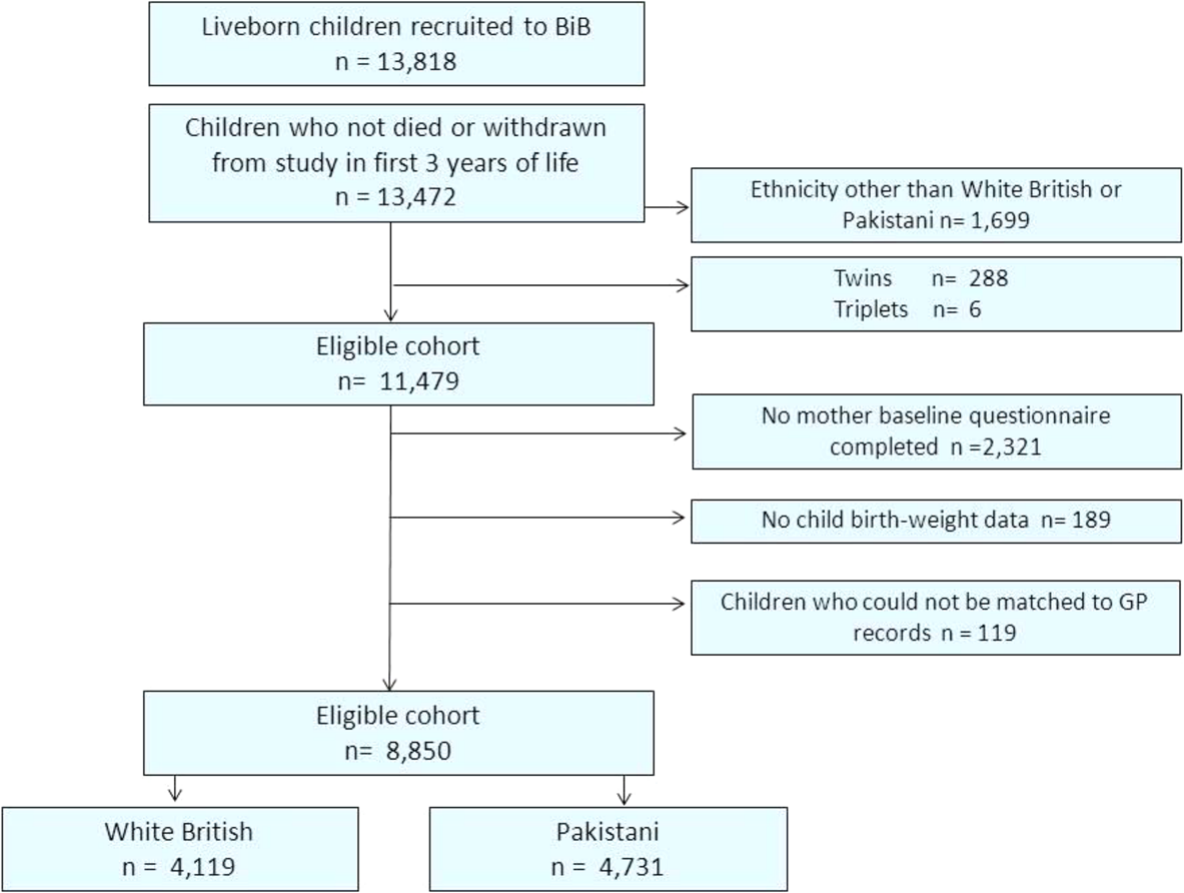
Study sample

The BiB study is a prospective birth cohort study that recruited women during pregnancy, full details of the study methodology have been previously reported [19]. To be eligible, women had to attend booking clinic between March 2007 and December 2010 and be booked to give birth in Bradford. Bradford is a city in the North of England with high levels of socioeconomic deprivation and ethnic diversity. Approximately half of the births in the city are to mothers of South Asian origin most of whom originate from Pakistan. Women were recruited to BiB at their 75 g oral glucose tolerance test (OGTT) appointment which is routinely offered at around 26–28 weeks gestation to all women booked for delivery in Bradford. Those who attended this appointment and agreed to take part in the study consented to the use of theirs and their child’s medical records, had their height and weight recorded and completed an interviewer administered questionnaire. The questionnaire included questions relating to ethnicity, social and economic circumstances, smoking, alcohol, diet, education, employment and place of birth. Interviews were conducted in a range of South Asian languages (including Mirpuri, Bengali, Punjabi). Mirpuri is the most commonly spoken Asian language in Bradford but has no written script therefore questionnaires were transliterated, that is translated verbally to Mirpuri and then written phonetically, precisely as spoken to ensure that all interpreters translated it in the same way. A total of 12,453 women who gave birth to 13,818 liveborn children were recruited to the study. For these analyses, multiple births, children born to parents of ethnic origin other than White British or Pakistani, children of mothers who did not complete a baseline questionnaire at recruitment, children with missing birthweight (for example those who were born outside the Bradford area) and children who could not be matched to their primary care record were all excluded (Fig. 1). Thus 8850 participants are included (4119 White British; 4731 Pakistani). Ethics approval for the study was provided by Bradford Local Research Ethics Committee (ref 06/Q1202/48).

### Outcome measurement

The number of general practice consultations and prescription data were derived from electronic records. Primary care electronic health records were obtained for BiB participants registered with GP surgeries that use the SystmOne platform. SystmOne has 100% coverage in Bradford and high coverage in surrounding areas. Records were extracted when NHS number, surname, date of birth and gender were an exact match in SystmOne. From the full BiB cohort of children, 99.0% were matched to their primary care records. Hospital episode statistics (HES) were obtained from the Health and Social Care Information Centre (HSCIC), matched to participants using the same process. Hospital admissions were categorised as Hospital Emergency (any emergency admission including to accident and emergency or direct to paediatric departments) and Hospital Elective which describes any elective admission either as an outpatient or inpatient (90% were outpatient episodes).

### Exposure measurement

Birthweight was obtained from hospital birth records and in all participants was recorded immediately following birth using SECA digital scales. We identified children as being born small using the World Health Organization (WHO) criteria for low birthweight as a weight at birth of below 2500 g. As a low birthweight can be the result of either premature birth or restricted growth in utero and because here we are primarily interested in restricted growth or low term birthweight, we included gestation as a covariable in the analyses of low birthweight (< 2500 g). We separately calculated customised birth weight centiles that take into account gestational age, maternal height, maternal pre-pregnancy or weight at booking, ethnicity, parity and neonatal sex (Gardosi 2004) and are recommended by the UK Royal College of Obstetrics and Gynaecology (RCOG) for assessment of birth weight [20]. SGA was defined as less than the tenth customised birth weight centile and all gestations were included in the customised chart analyses. Duration of gestation was obtained from hospital birth records and was based on the date of the mother’s last menstrual period which was confirmed by a dating ultrasound at around 12 weeks.

### Assessment of ethnicity

Ethnicity was self-reported at the mother’s questionnaire interview and based on UK Office of National Statistics guidance details of which have been previously reported [21]. For these analyses, children were defined as White British or Pakistani origin.

### Covariables

A priori we considered maternal parity, infant sex, gestational age, maternal age, social economic information (maternal education, housing tenure, means-tested benefits) and smoking as characteristics that might confound any associations. Maternal parity, gestational age (to the last completed week) and infant sex were all obtained from obstetric medical records. Customised birthweight charts account for gestation, parity and infant sex therefore these variables were only added to low birthweight analyses. Maternal age, social economic information (maternal education, housing tenure, means-tested benefits) and smoking data were obtained from the interviewer administered mother’s questionnaire completed at recruitment. We equivalised the mother’s highest educational qualifications (based on the qualification received and the country obtained) into one of several categories using UK NARIC (http://www.ecctis.co.uk/naric/default.aspx): *< 5 GCSE equivalent, ≥5 GCSE equivalent, ‘A’ level equivalent, Higher than A-level equivalent, Other qualifications (*e.g. *City and Guilds, RSA/OCR, BTEC), Don’t know, Foreign Unknown.* Don’t know relates to the mother responding “don’t know” during interview. Foreign Unknown relates to a qualification listed in the free text response but no level of qualification is given or the qualification listed cannot be equivalised to one of the above categories. For these analyses, women were categorised as having been educated beyond the age of 18 or not (i.e. *Higher than A-level equivalent, Other qualifications (*e.g. *City and Guilds, RSA/OCR, BTEC),university undergraduate courses)*. Don’t know and Unknown were categorized as not educated beyond the age of 18. Receipt of means tested benefits was based on the mother or her household receiving any of: Income Support, Job Seekers Allowance, Working Tax Credit or Housing Benefit. Housing tenure was categorised according to whether the woman lived in a household where the home was either part-owned (i.e. mortgaged) or owned outright, or not (i.e. rented). Maternal smoking was categorised as never, past (but not during this index pregnancy), current/during the index pregnancy.

### Statistical analyses

All analyses were performed using Stata (version 13). Negative binomial regression models were employed as the outcome measures (counts of GP consultation rates, prescriptions and hospital episodes) were over dispersed and did not fit a Poisson distribution well. Models were constructed for each outcome and used to predict the incidence of GP consultations, number of prescriptions and hospital episodes for children based on their ethnicity, low birthweight and SGA categories: after adjusting for the covariables described above and taking into account individual exposure time (the proportion of the study period that a child is registered with a GP practice using SystemOne). Incidence rate ratios (IRR), the ratio of predicted events for Pakistani children compared to White British children, with 95% confidence intervals (CI) were also derived to aid the substantive interpretation of ethnic differences.

## Results

Table 1 shows maternal and child characteristics for all participants and by ethnic group. Maternal age and education level were similar in both ethnic groups. A higher proportion of Pakistani mothers lived in owner-occupied housing and received means tested benefits than White British women. They were on average shorter, lighter and had a lower BMI than White British mothers. Smoking was markedly less common among Pakistani origin women of whom 92.1% reported having never smoked compared to 41.7% of White British women. Pakistani children had a lower mean birthweight and a higher proportion had a low birthweight defined as less than 2500 g compared to White British children (8.7 and 5% respectively). Using customised growth charts, the proportion of Pakistani children classified as SGA was 15.3% compared to 6.2% of White British children. Sex, gestational age and pre-term births were similar in both groups (5.3% of White British children and 4.6% of Pakistani children were born at less than 37 weeks gestation). Incidence of all markers of morbidity was higher among Pakistani children compared to White British children with the exception of bronchodilator prescriptions. On average, Pakistani origin children had 23.5 (standard deviation (SD) 13.8) GP appointments compared to 16 (SD 11.6) among White British children and had a higher number of antibiotic and markedly higher analgesic prescriptions. Hospital episodes were uncommon in both groups but more common among Pakistani children compared to White British children and this was the case for both emergency (0.45 (SD 1.12) and 0.38 (SD 0.83) respectively) and elective admissions (0.10 (SD 0.77) and 0.07 (SD 0.36)).

**Table 1:** Maternal and child characteristics overall and by ethnic group, n (%) or mean (SD), with *p* values for the difference between White British and Pakistani participants (2 sided t test or chi-squared test)

	All	White British	Pakistani	*p* value
	8850	4119	4731	
Mother variables				
Mother age at delivery	27.5 (5.61)	27.0 (6.05)	27.9 (5.16)	< 0.001
Mother education higher than A level	2540 (28.7%)	1162 (28.2%)	1372 (29.0%)	= 0.420
Housing tenure (Owner occupied)	5487 (62.0%)	2175 (52.8%)	3312 (70.0%)	< 0.001
Household in receipt of means tested benefits	3797 (42.9%)	1557 (37.8%)	2242 (47.4%)	< 0.001
Mother height (cm)	161.8 (6.35)	164.1 (6.23)	159.7 (5.72)	< 0.001
Mother weight (kg)	74.3 (15.6)	77.9 (16.5)	71.1 (14.0)	< 0.001
Mother BMI (at recruitment)	28.3 (5.47)	28.9 (5.72)	27.8 (5.19)	< 0.001
Parity 0	3513 (39.7%)	4283 (48.4%)	2841 (32.1%)	< 0.001
Parity 1	2558 (28.9%)	2788 (31.5%)	2354 (26.6%)	
Parity 2+	2779 (31.4%)	1779 (20.1%)	3655 (41.3%)	
Mother never smoked	6071 (68.6%)	3690 (41.7%)	8151 (92.1%)	< 0.001
Mother smoked before pregnancy	1478 (16.7%)	2673 (30.2%)	434 (4.9%)	
Mother smoked during pregnancy	1301 (14.7%)	2496 (28.2%)	266 (3.0%)	
Child Variables				
Birth-weight (g)	3238 (541)	3371 (547)	3147 (518)	< 0.001
Low birthweight (< 2500 g)	620 (7.0%)	443 (5.0%)	770 (8.7%)	< 0.001
Small For Gestational Age (GROW – 10th Decile)	902 (15.8%)	257 (6.24%)	645 (15.3%)	< 0.001
Gestation (weeks)	39.2 (1.72)	39.3 (1.76)	39.2 (1.68)	= 0.006
Pre-term birth (< 37 weeks)	439 (5.0%)	220 (5.3%)	219 (4.6%)	= 0.124
Gender (female)	4319 (48.8%)	4283 (48.4%)	4345 (49.1%)	= 0.542
GP appointments	20.0 (13.3)	16.0 (11.6)	23.5 (13.8)	< 0.001
Analgesic prescriptions	4.65 (4.97)	2.82 (3.10)	6.24 (5.68)	< 0.001
Antibiotic prescriptions	3.24 (3.90)	2.64 (3.26)	3.77 (4.32)	< 0.001
Bronchodilator prescriptions	1.44 (3.18)	1.49 (3.04)	1.40 (3.29)	= 0.184
Emergency hospital episodes	0.42 (1.00)	0.38 (0.83)	0.45 (1.12)	= 0.001
Elective hospital episodes	0.08 (0.61)	0.07 (0.36)	0.10 (0.77)	= 0.022

Being born small whether classified as low birth-weight (< 2500 g) or SGA, was generally associated with an increased rate of all outcomes compared to children not born small and this was consistent across both ethnic groups (Table 2), although the magnitude of difference varied between outcomes and between ethnic groups. There was a significant ethnic difference across all birthweight categories for GP appointments and analgesic prescriptions with markedly higher rates among Pakistani origin children. There were also ethnic differences in antibiotic prescriptions and Hospital Emergency and Hospital Elective episodes among normal weight children irrespective of how that was categorised.

**Table 2:** Adjusted^a^ incidence rate (95% CI) per person year by ethnicity and birthweight category (defined using low birth-weight criteria of birthweight < 2500 g and by customized growth centiles (SGA))

	White British *N* = 4119	Pakistani *N* = 4731
GP appointments Rate per person year		
Not Low birthweight	15.8 (15.4–16.2)	23.7 (23.1–24.2)
Low birthweight	17.9 (15.9–19.9)	26.5 (24.5–28.4)
Not SGA	15.8 (15.4–16.2)	23.5 (23.0–24.1)
SGA	16.4 (14.7–18.0)	25.3 (23.9–26.7)
Analgesic prescriptions Rate per person year		
Not low birthweight	2.75 (2.64–2.85)	6.35 (6.14–6.57)
Low birthweight	3.53 (2.87–4.18)	6.22 (5.53–6.91)
Not SGA	2.74 (2.64–2.85)	6.30 (6.08–6.53)
SGA	3.39 (2.81–3.98)	6.51 (5.93–7.09)
Antibacterial prescriptions Rate per person year		
Not low birthweight	2.63 (2.52–2.74)	3.63 (3.50–3.76)
Low birthweight	3.45 (2.77–4.13)	3.91 (3.45–4.37)
Not SGA	2.63 (2.52–2.74)	3.64 (3.50–3.78)
SGA	2.89 (2.35–3.44)	3.65 (3.29–4.01)
Bronchodilator prescriptions Rate per person year		
Not low birthweight	1.43 (1.30–1.55)	1.36 (1.25–1.48)
Low birthweight	1.99 (1.19–2.79)	2.03 (1.46–2.60)
Not SGA	1.42 (1.30–1.55)	1.37 (1.25–1.49)
SGA	1.38 (0.81–1.95)	1.52 (1.14–1.91)
Hospital Emergency Rate per person year		
Not low birthweight	0.35 (0.32–0.38)	0.44 (0.40–0.47)
Low birthweight	0.56 (0.37–0.75)	0.80 (0.61–0.99)
Not SGA	0.35 (0.32–0.38)	0.42 (0.39–0.45)
SGA	0.40 (0.26–0.55)	0.59 (0.47–0.71)
Hospital Elective Rate per 100 person years		
Not low birthweight	5.77 (4.58–6.96)	9.54 (7.80–11.27)
Low birthweight	22.80 (8.75–36.85)	13.91 (7.88–19.93)
Not SGA	5.36 (4.26–6.46)	7.99 (6.49–9.49)
SGA	22.52 (1.74–43.31)	18.96 (10.77–27.15)

^a^Low birthweight models adjusted for maternal parity, infant sex, gestational age, maternal age, social economic factors (maternal education, housing tenure, means- tested benefits) and smoking; SGA models adjusted for maternal age, social economic factors (maternal education, housing tenure, means- tested benefits) and smoking

**Fig. 2:**
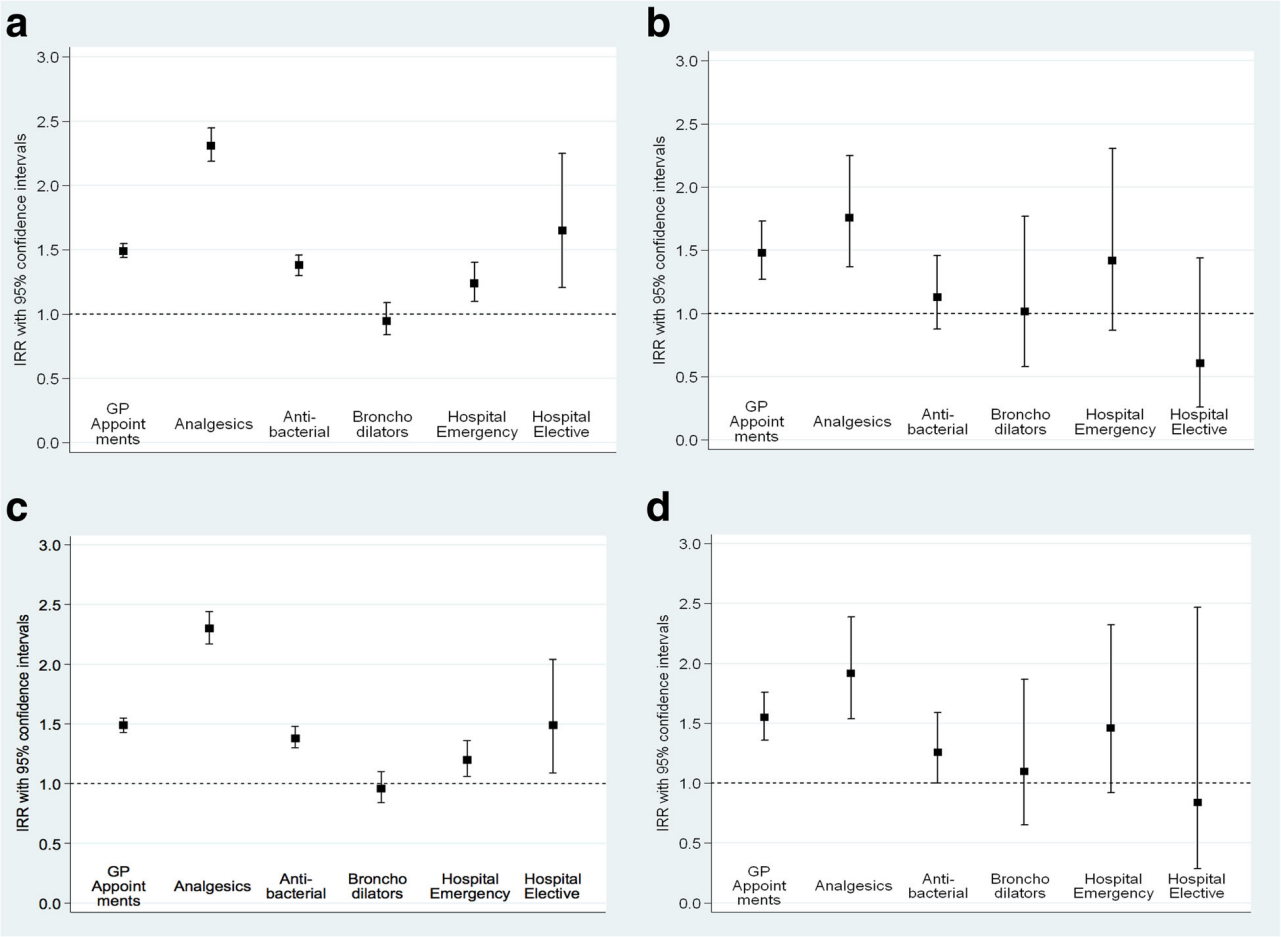
**a** Adjusted Incident Rate Ratio (IRR) for children of Pakistani mothers. (Baseline group is children of White British mothers = 1). Children who are not low birth-weight (i.e. 2500 g or more). *Low birthweight models adjusted for maternal parity, infant sex, gestational age, maternal age, social economic factors (maternal education, housing tenure, means- tested benefits) and smoking; SGA models adjusted for maternal age, social economic factors (maternal education, housing tenure, means- tested benefits) and smoking. **b** Adjusted Incident Rate Ratio (IRR) for children of Pakistani mothers (Baseline group is children of White British mothers = 1). For children who are low birth-weight (i.e. less than 2500 g). *Low birthweight models adjusted for maternal parity, infant sex, gestational age, maternal age, social economic factors (maternal education, housing tenure, means- tested benefits) and smoking; SGA models adjusted for maternal age, social economic factors (maternal education, housing tenure, means- tested benefits) and smoking. **c** Adjusted Incident Rate Ratio (IRR) for children of Pakistani mothers (Baseline group is children of White British mothers = 1). For children who are not small for gestational age (SGA- GROW). *Low birthweight models adjusted for maternal parity, infant sex, gestational age, maternal age, social economic factors (maternal education, housing tenure, means- tested benefits) and smoking; SGA models adjusted for maternal age, social economic factors (maternal education, housing tenure, means- tested benefits) and smoking. **d** Adjusted Incident Rate Ratio (IRR) for children of Pakistani mothers (Baseline group is children of White British mothers = 1). For children who are small for gestational age (SGA- GROW). *Low birthweight models adjusted for maternal parity, infant sex, gestational age, maternal age, social economic factors (maternal education, housing tenure, means- tested benefits) and smoking; SGA models adjusted for maternal age, social economic factors (maternal education, housing tenure, means- tested benefits) and smoking

Table 3 and Fig. 2 a – d show the adjusted IRRs for Pakistani children relative to White British children for each marker of morbidity. Pakistani children generally had a higher rate of episodes for all outcomes compared to White British children whether they were normal weight or categorised as small at birth by either method. They had 48–55% more GP appointments depending on the birthweight category, compared to White British children. The IRR for Pakistani children relative to White British children for analgesic prescriptions ranged from 1.76 (95% CI 1.37, 2.25) to 2.31 (95% CI 2.19, 2.45) across the categories of normal and small birthweight and antibiotic prescriptions ranged from 13 to 38% higher among Pakistani origin children. Compared to White British children, bronchodilator prescriptions were slightly more common among Pakistani children categorised as being small at birth (by either method) compared to White British children, although there was no strong statistical evidence for this difference. The incidence of Hospital Emergency episodes was greater among Pakistani children and especially where children were born small although again, these results were not statistically significant which in this case, mostly reflects the small number of emergency episodes overall. The incidence of Hospital Elective episodes was markedly higher among Pakistani origin children compared to White British children where they were classified as normal weight using either the 2500 g cut-off or the customised charts (IRR 1.65 (95% CI 1.21–2.25) and 1.49 (95% CI 1.09, 2.04) respectively). However, among children born small the IRR was 0.61 (95% CI 0.26, 1.44) for children categorised as low birthweight (< 2500 g) and 0.84 (95% CI 0.29, 2.47) for those defined small using the customised charts.

**Table 3:** Adjusted^a^ incidence rate ratio (95% CI) by ethnicity and birthweight category; (defined using low birth-weight criteria of birthweight < 2500 g and by customized growth centiles (SGA))

GP appointments	White British n(%)	Pakistani n(%)	Incidence Rate Ratio Pakistani/ White British
Not low birthweight	3913 (95.0%)	4319 (91.3%)	1.49 (1.44–1.55)
Low birthweight	206 (5.0%)	412 (8.7%)	1.48 (1.27–1.73)
Not SGA	3658 (93.8%)	3899 (86.4%)	1.49 (1.43–1.55)
SGA	241 (6.2%)	613 (13.6%)	1.55 (1.36–1.76)
Analgesic prescriptions			
Not low birthweight	3913 (95.0%)	4319 (91.3%)	2.31 (2.19–2.45)
Low birthweight	206 (5.0%)	412 (8.7%)	1.76 (1.37–2.25)
Not SGA	3658 (93.8%)	3899 (86.4%)	2.30 (2.17–2.44)
SGA	241 (6.2%)	613 (13.6%)	1.92 (1.54–2.39)
Antibiotic prescriptions			
Not low birthweight	3913 (95.0%)	4319 (91.3%)	1.38 (1.30–1.46)
Low birthweight	206 (5.0%)	412 (8.7%)	1.13 (0.88–1.46)
Not SGA	3658 (93.8%)	3899 (86.4%)	1.38 (1.30–1.48)
SGA	241 (6.2%)	613 (13.6%)	1.26 (1.00–1.59)
Bronchodilator prescriptions			
Not low birthweight	3913 (95.0%)	4319 (91.3%)	0.95 (0.84–1.09)
Low birthweight	206 (5.0%)	412 (8.7%)	1.02 (0.58–1.77)
Not SGA	3658 (93.8%)	3899 (86.4%)	0.96 (0.84–1.10)
SGA	241 (6.2%)	613 (13.6%)	1.10 (0.65–1.87)
Hospital Emergency			
Not low birthweight	3913 (95.0%)	4319 (91.3%)	1.24 (1.10–1.40)
Low birthweight	206 (5.0%)	412 (8.7%)	1.42 (0.87–2.31)
Not SGA	3658 (93.8%)	3899 (86.4%)	1.20 (1.06–1.36)
SGA	241 (6.2%)	613 (13.6%)	1.46 (0.92–2.32)
Hospital Elective			
Not low birthweight	3913 (95.0%)	4319 (91.3%)	1.65 (1.21–2.25)
Low birthweight	206 (5.0%)	412 (8.7%)	0.61 (0.26–1.44)
Not SGA	3658 (93.8%)	3899 (86.4%)	1.49 (1.09–2.04)
SGA	241 (6.2%)	613 (13.6%)	0.84 (0.29–2.47)

^a^Low birthweight models adjusted for maternal parity, infant sex, gestational age, maternal age, social economic factors (maternal education, housing tenure, means- tested benefits) and smoking; SGA models adjusted for maternal age, social economic factors (maternal education, housing tenure, means- tested benefits) and smoking

In all analyses, results were generally similar whether small at birth was defined using the cut-off for low birthweight of a birthweight less than 2500 g or defined using the customised SGA charts. Adjustment for social economic variables did not markedly alter the results (see Additional file 1: Tables S1 and S2).

## Discussion

To our knowledge, this is the first time that detailed research information has been linked to primary and secondary care outcome data to examine the association between being born small and early childhood morbidity. Consistent with previous results using this cohort [13, 22] and other UK studies [12, 23], Pakistani children had a lower mean birthweight and were more likely to have a low birthweight (< 2500 g) compared to White British children. Being born small has previously been associated with an increased risk of adverse neonatal outcomes [24] and there is some association with adult morbidity [6, 25] although this is possibly modified by adult risk factors, for example adult BMI [26]. There is however, a notable lack of evidence to identify whether similar associations are present in childhood. We used GP consultations, prescription data for the three most common prescriptions, and hospital episode information as markers of morbidity in a cohort of children all born and growing up in the same UK city. We found that in both ethnic groups, children who were born small, regardless of how that was categorised (i.e. either low birthweight or SGA), had a higher incidence of most markers of morbidity from birth to age 3, compared to children of normal weight (i.e. not low birthweight or SGA). This suggests that the association of being born small and poorer health outcomes identified in the neonatal period [24] may persist into early childhood and underlines how prevention of low birthweight remains important to the development of public health interventions.

We found that compared to White British children, Pakistani children had a higher incidence of all morbidity markers with the exception of bronchodilator prescriptions and Hospital Elective episodes. This greater health service use among Pakistani origin children is consistent with other studies that report higher rates of GP consultations [27], and hospital emergency admissions [28, 29] among ethnic minority groups. It has previously been suggested that these differences might in part be explained by social and economic differences between groups, however our results did not differ substantially with or without adjustment for social and economic markers (Additional file 1: Tables S1 and S2). In contrast to previous evidence of higher rates of antibiotic prescriptions among UK white populations across all age groups [30], we found antibiotic prescriptions were more common for Pakistani children compared to White British children across all birthweight categories but especially among normal weight children (defined by either method) where the rate of prescriptions was up to 38% higher. Given the current concerns around antibiotic use [31], these population differences require further investigation and may be a potential area for future targeted interventions to protect the population’s health. Pakistani children also had on average, a greater number of prescriptions for analgesics, which were the most common prescription category in both ethnic groups and may reflect current UK guidance for optimising analgesia for the treatment of childhood infections [32]. We found that bronchodilator prescriptions were slightly more common in White British children despite previous findings from this cohort that identified a higher proportion of Pakistani children as being diagnosed with asthma compared to White British children (13.4% compared to 8.9%) [33]. Our results are consistent with other studies that have identified differences in specialty asthma care and higher rates of related emergency admissions among Pakistani populations [29, 34] and also with the wider possibility that minority ethnic groups may receive less preventive health care which leads to greater use of emergency care [34]. Our finding that emergency episodes were more common among Pakistani children across all birthweight categories seems to support this. Similarly, previous studies have identified that South Asians are more likely to consult their GP but are less likely to be referred to secondary care [27, 35]. Here, our results are partly consistent with this in that we found that Pakistani children had a higher rate of GP consultations in all birthweight categories but that the rate of elective episodes was only lower for those children defined as small at birth. In normal birthweight children, the rate of elective episodes was on average higher than that for White British children. GP decision making is likely to be independent of birthweight therefore rather than being suggestive of ethnic differences in elective referral, fewer elective episodes among Pakistani children defined as small might suggest that some of these children are born small and healthy as opposed to small and at risk thus needing less elective care. However, the rate of elective episodes was lower for those Pakistani children born small even when customised growth charts were applied, our other markers of morbidity do not support the possibility of less morbidity in these children and our estimates are based on a small number of elective episodes and the confidence limits for the ethnic difference suggest some uncertainty.

We used two methods to identify children who were born small. First, we used a cut-off of birthweight less than 2500 g as although this is a crude measure [36], it is well recognised and established as an indicator of health [37]. Second, we used GROW customised birthweight charts which adjust for ethnicity and maternal characteristics (height, BMI, age and parity). In our study population, we found that when using the cut-off of birthweight < 2500 g, 8.7% of Pakistani children and 5% of White British were defined as low birthweight. When we applied the customised charts, more children in both ethnic groups (13.6 and 6.2% respectively) were defined as SGA compared to the number who were categorised as low birthweight (< 2500 g) but this was especially marked among Pakistani children. Despite being intuitively appealing, customised charts have not improved the prediction of growth restriction [18] or adverse neonatal outcomes [38, 39] compared with population standard charts. Here, whilst there are some differences in outcomes between low birthweight and SGA definitions, we have found no robust evidence that customised charts better predict early childhood morbidity than a crude cut-off of being born weighing less than 2500 g.

The key strengths of this study are our linkage of research data with routine primary and secondary care data which has allowed us to examine the effects of being born small on child health beyond the perinatal period, our detailed ethnicity information and the ability to adjust for a range of covariables. A limitation of our study is that our outcomes may not accurately reflect morbidity for a number of reasons. First, the data are dependent on the accuracy and quality of coding. Second, for hospital episodes the sample numbers are small in some categories and this is evident in the confidence intervals for these outcomes. Third, GP consultations include routine appointments (for example immunisations) that may not be indicative of illness however, we expect this to not differ markedly between the two ethnic groups. Over 99% of analgesic prescriptions were paracetamol or paracetamol based and it is possible that some of these prescriptions may have been associated with routine immunization but we were unable to examine this with the data available, however if this is the case we do not expect it would differ markedly between the two groups’. Likewise, Hospital Emergency episodes will include accident related episodes that do not necessarily reflect morbidity, it is possible that these may differ between the two groups but we are not able to examine this further with the data we have available. Children born prematurely might have a greater risk of respiratory illness and wheeze and as such experience greater morbidity in early childhood [40], however prematurity did not differ markedly between the two ethnic groups and gestational age was accounted for in all models whether low birthweight was defined as less than 2500 g or using customised growth charts. In addition, we were only able to examine Pakistani origin children due to the small number of other South Asian groups in the BiB cohort. This means that our results may not be generalisable to other South Asian groups.

## Conclusion

These results suggest that being categorised as small at birth is associated with increased morbidity estimated using health service use information, in early childhood in both White British and Pakistani origin UK children. This combined with evidence that birthweight is inversely associated with neonatal mortality, educational achievement and adult disease risk [2–6], highlights the importance of birthweight to health throughout the life-course and that the development of interventions to reduce low birthweight, remains a public health priority. Overall, Pakistani children access primary and secondary health services more frequently and are more commonly prescribed analgesics and antibiotics than White British children irrespective of whether they are born small or how this is defined. This has implications for health service planning in areas with large South Asian populations and suggests a need for a better understanding of ethnic differences in health service use. Despite the marked difference in the criteria used to define low birthweight and SGA, we found our results did not differ substantially using either method which supports the suggestion that customised charts do not necessarily better predict outcomes.

## Additional files

Additional file 1: **Table S1.** Comparison of models of predicted child outcome measures (with 95% CI), by ethnicity, low birth-weight and small for gestational age (SGA-GROW) with and without adjustment for socio-economic variables (predicted rates with 95% CI). **Table S2.** Comparison of adjusted* incidence rate ratios (95% CI) of child outcome measures by ethnicity, low birth-weight and small for gestational age (SGA-GROW) with and without adjustment for socioeconomic variables. (DOC 89 kb)

## Abbreviations

BiB: Born in Bradford
CI: Confidence interval
GP: General Practitioner
HES: Hospital Episode Statistics
IRR: Incidence rate ratio
OGTT: Oral glucose tolerance test
RCOG: Royal College of Obstetricians and Gynaecologists
SD: Standard deviation
SGA: Small for gestational age
