# Supplementary material for: Is small size at birth associated with early childhood morbidity in white British and Pakistani origin UK children aged 0–3? Findings from the born in Bradford cohort study
Source: BMC Pediatr. 2018 Feb 1;18:22. doi: 10.1186/s12887-018-0987-0 (PMC5796403; doi:10.1186/s12887-018-0987-0)
Supplement: Supplementary file 1 — Table S1. Comparison of models of predicted child outcome measures (with 95% CI), by ethnicity, low birth-weight and small for gestational age (SGA-GROW) with and without adjustment for socio-economic variables (predicted rates with 95% CI). Table S2. Comparison of adjusted* incidence rate ratios (95% CI) of child outcome measures by ethnicity, low birth-weight and small for gestational age (SGA-GROW) with and without adjustment for socioeconomic variables. (DOC 89 kb) [file 12887_2018_987_MOESM1_ESM.doc]

Supplementary Table 1

**Comparison of models of predicted child outcome measures (with 95% CI), by ethnicity, low birth-weight and small for gestational age (SGA-GROW) with and without adjustment for socio-economic variables (predicted rates with 95% CI).**

|  | **White British (without socioeconomic controls)** | **Pakistani (without socioeconomic controls)** | **White British (with socioeconomic controls)** | **Pakistani (with socioeconomic controls)** |
| --- | --- | --- | --- | --- |
|  | **N = 4,119** | **N = 4,731** | **N = 4,119** | **N = 4,731** |
| **GP appointments** Rate per person year | | |  | |
| Not Low birthweight | 15.8 (15.4-16.2) | 23.7 (23.2-24.3) | 15.8 (15.4-16.2) | 23.7 (23.1-24.2) |
| Low birthweight | 17.7 (15.7-19.7) | 26.6 (24.6-28.6) | 17.9 (15.9-19.9) | 26.5 (24.5-28.4) |
| Not SGA | 15.8 (15.4-16.2) | 23.6 (23.0-24.2) | 15.8 (15.4-16.2) | 23.5 (23.0-24.1) |
| SGA | 16.3 (14.7-18.0) | 25.4 (24.0-26.8) | 16.4 (14.7-18.0) | 25.3 (23.9-26.7) |
| **Analgesic prescriptions** Rate per person year | | |  | |
| Not low birthweight | 2.77 (2.66-2.87) | 6.33 (6.12-6.54) | 2.75 (2.64-2.85) | 6.35 (6.14-6.57) |
| Low birthweight | 3.48 (2.85-4.11) | 6.28 (5.58-6.99) | 3.53 (2.87-4.18) | 6.22 (5.53-6.91) |
| Not SGA | 2.76 (2.65-2.87) | 6.29 (6.07-6.51) | 2.74 (2.64-2.85) | 6.30 (6.08-6.53) |
| SGA | 3.43 (2.85-4.02) | 6.50 (5.93-7.08) | 3.39 (2.81-3.98) | 6.51 (5.93-7.09) |
| **Antibacterial prescriptions** Rate per person year | | |  | |
| Not low birthweight | 2.61 (2.51-2.72) | 3.66 (3.52-3.80) | 2.63 (2.52-2.74) | 3.63 (3.50-3.76) |
| Low birthweight | 3.42 (2.74-4.09) | 3.96 (3.50-4.43) | 3.45 (2.77-4.13) | 3.91 (3.45-4.37) |
| Not SGA | 2.61 (2.50-2.72) | 3.67 (3.52-3.81) | 2.63 (2.52-2.74) | 3.64 (3.50-3.78) |
| SGA | 2.86 (2.32-3.41) | 3.68 (3.32-4.04) | 2.89 (2.35-3.44) | 3.65 (3.29-4.01) |
| **Bronchodilator prescriptions** Rate per person year | | |  | |
| Not low birthweight | 1.41 (1.29-1.54) | 1.38 (1.26-1.49) | 1.43 (1.30-1.55) | 1.36 (1.25-1.48) |
| Low birthweight | 1.94 (1.16-2.72) | 2.07 (1.48-2.66) | 1.99 (1.19-2.79) | 2.03 (1.46-2.60) |
| Not SGA | 1.41 (1.28-1.53) | 1.39 (1.27-1.51) | 1.42 (1.30-1.55) | 1.37 (1.25-1.49) |
| SGA | 1.38 (0.81-1.95) | 1.52 (1.14-1.91) | 1.38 (0.81-1.95) | 1.52 (1.14-1.91) |
| **Hospital Emergency** Rate per person year | | |  | |
| Not low birthweight | 0.35 (0.32-0.38) | 0.43 (0.40-0.47) | 0.35 (0.32-0.38) | 0.44 (0.40-0.47) |
| Low birthweight | 0.55 (0.36-0.74) | 0.80 (0.61-0.99) | 0.56 (0.37-0.75) | 0.80 (0.61-0.99) |
| Not SGA | 0.35 (0.32-0.38) | 0.42 (0.39-0.45) | 0.35 (0.32-0.38) | 0.42 (0.39-0.45) |
| SGA | 0.41 (0.26-0.56) | 0.58 (0.47-0.70) | 0.40 (0.26-0.55) | 0.59 (0.47-0.71) |
| **Hospital Elective** Rate per 100 person years | | |  | |
| Not low birthweight | 5.62 (4.47-6.77) | 9.71 (7.94-6.77) | 5.77 (4.58-6.96) | 9.54 (7.80-11.27) |
| Low birthweight | 21.86 (8.49-35.22) | 13.95 (7.85-20.05) | 22.80 (8.75-36.85) | 13.91 (7.88-19.93) |
| Not SGA | 5.26 (4.19-6.32) | 8.07 (6.56-9.59) | 5.36 (4.26-6.46) | 7.99 (6.49-9.49) |
| SGA | 20.22 (8.89-39.55) | 19.32 (11.31-27.34) | 22.52 (1.74-43.31) | 18.96 (10.77-27.15) |

*Low birthweight models adjusted for maternal parity, infant sex, gestational age, maternal age, social economic factors (maternal education, housing tenure, means- tested benefits) and smoking; SGA models adjusted for maternal age, social economic factors (maternal education, housing tenure, means- tested benefits) and smoking

Supplementary Table 2

**Comparison of adjusted* incidence rate ratios (95% CI) of child outcome measures by ethnicity, low birth-weight and small for gestational age (SGA-GROW) with and without adjustment for socioeconomic variables**

| **GP appointments** | **Incidence Rate Ratio Pakistani/ White British** | |
| --- | --- | --- |
|  | **Without socioeconomic controls** | **With socioeconomic controls** |
| Not low birthweight | 1.50 (1.45-1.56) | 1.49 (1.44-1.55) |
| Low birthweight | 1.51 (1.29-1.76) | 1.48 (1.27-1.73) |
| Not SGA | 1.50 (1.44-1.56) | 1.49 (1.43-1.55) |
| SGA | 1.55 (1.36-1.76) | 1.55 (1.36-1.76) |
| **Analgesic prescriptions** |  |  |
| Not low birthweight | 2.29 (2.17-2.42) | 2.31 (2.19-2.45) |
| Low birthweight | 1.81 (1.41-2.31) | 1.76 (1.37-2.25) |
| Not SGA | 2.28 (2.15-2.42) | 2.30 (2.17-2.44) |
| SGA | 1.92 (1.54-2.39) | 1.92 (1.54-2.39) |
| **Antibiotic prescriptions** |  |  |
| Not low birthweight | 1.40 (1.32-1.49) | 1.38 (1.30-1.46) |
| Low birthweight | 1.16 (0.90-1.50) | 1.13 (0.88-1.46) |
| Not SGA | 1.40 (1.32-1.50) | 1.38 (1.30-1.48) |
| SGA | 1.26 (1.00-1.60) | 1.26 (1.00-1.59) |
| **Bronchodilator prescriptions** | | |
| Not low birthweight | 0.97 (0.85-1.11) | 0.95 (0.84-1.09) |
| Low birthweight | 1.07 (0.61-1.87) | 1.02 (0.58-1.77) |
| Not SGA | 0.99 (0.86-1.13) | 0.96 (0.84-1.10) |
| SGA | 1.10 (0.65-1.87) | 1.10 (0.65-1.87) |
| **Hospital Emergency** |  |  |
| Not low birthweight | 1.23 (1.09-1.39) | 1.24 (1.10-1.40) |
| Low birthweight | 1.45 (0.89-2.36) | 1.42 (0.87-2.31) |
| Not SGA | 1.18 (1.04-1.34) | 1.20 (1.06-1.36) |
| SGA | 1.46 (0.92-2.32) | 1.46 (0.92-2.32) |
| **Hospital Elective** |  |  |
| Not low birthweight | 1.73 (1.27-2.35) | 1.65 (1.21-2.25) |
| Low birthweight | 0.64 (0.27-1.52) | 0.61 (0.26-1.44) |
| Not SGA | 1.54 (1.12-2.10) | 1.49 (1.09-2.04) |
| SGA | 0.84 (0.29-2.47) | 0.84 (0.29-2.47) |

*Low birthweight models adjusted for maternal parity, infant sex, gestational age, maternal age, social economic factors (maternal education, housing tenure, means- tested benefits) and smoking; SGA models adjusted for maternal age, social economic factors (maternal education, housing tenure, means- tested benefits) and smoking
